# Genome Sequence of the Quorum-Quenching *Agrobacterium tumefaciens* Strain WRT31

**DOI:** 10.1128/genomeA.00653-13

**Published:** 2013-08-22

**Authors:** Samuel Mondy, Ouaghlis Lalouche, Yves Dessaux, Denis Faure

**Affiliations:** Institut des Sciences du Végétal, CNRS, Gif-sur-Yvette, France

## Abstract

*Agrobacterium tumefaciens* strain WRT31 is a quorum-sensing signal-degrading bacterium that has been isolated from the rhizosphere of tobacco plants. This strain belongs to *A. tumefaciens* genomovar G1, is avirulent on various putative host plants, devoid of Ti plasmid, and contains the *blcC* gene encoding a gamma-butyrolactonase.

## GENOME ANNOUNCEMENT

Among the cultured community collected from tobacco rhizosphere, *Agrobacterium tumefaciens* strain WRT31 has been identified as an isolate that efficiently degrades the quorum-sensing (QS) signals of the *N*-acyl homoserine lactone (AHL) class (1, 2). This strain is avirulent on different hosts (*Datura stramonium* and tomato plants). It is defective for the plasmid Ti, and hence for the *traI* gene that encodes the synthesis of the QS signal 3-oxo-octanoyl-homoserine lactone (3-OC8-HSL). However, strain WRT31 harbors the lactonase-encoding gene *blcC* (*attM*) that is involved in the assimilation of gamma-butyrolactone as a carbon source and the cleavage of AHL QS signals, including 3-OC8-HSL (3, 4). In strain WRT31, cleavage of the AHL QS signals occurred even in the absence of known *blcC* inducers, such as gamma-aminobutyric acid, gamma-butyrolactone, and gamma-hydroxybutyric acid (4–6). This feature might suggest a constitutive expression of the lactonase BlcC or the presence of uncharacterized additional quorum-quenching enzymes in WRT31, hence its efficient AHL degradation ability.

Here, we report the *de novo* genome assembly of *A. tumefaciens* strain WRT31. Two libraries were constructed using the TruSeq SBS v3 sequencing kit: a shotgun (SG) paired-end library with a fragment size between 150 and 500 bp and a long jumping distance (LJD) mate-pair library with an average insert size of 7,765 bp. The two libraries were sequenced using a 2 × 100 bp paired-end read module of Illumina HiSeq 2000 by Eurofins Genomics (France). Sequence reads with low quality (<0.05), ambiguous nucleotides (*n* > 1), and a sequence length of <50 nucleotides were discarded prior to assembly. After trimming, 40,155,546 paired-end reads were retained (3,786,667,988 bases) with an average length of 94.3 bp, and 7,117,455 mate-paired reads (590,748,765 bases) with an average length of 83 bp. Sequence assembly was carried out using the CLC Genomics Workbench v5.5 (CLC bio, Aarhus, Denmark), with a read length of 0.5 and a similarity of 0.8. Nineteen contigs were obtained, with lengths ranging from 2 kbp to 844 kbp and an N_50_ value of 596,214 bp. The scaffolding was processed using SSPACE basic v2.0 (7). The *in silico* finishing of some gaps was carried out by mapping (read length of 0.9 and similarity of 0.95) the mate pair reads on each of the 5-kbp contigs ends. The collected reads were used for *de novo* local assembling (read length of 0.5 and similarity of 0.8). The published sequence is composed of 10 contigs (from 2 kbp to 2.17 Mbp) grouped in 4 scaffolds, with a coverage rate ranging from 569- to 827-fold.

The *A. tumefaciens* WRT31 genome consists of one circular chromosome containing 2,938,081 bp, one linear chromosome containing 2,176,692 bp, and two additional scaffolds containing 674,326 bp and 86,649 bp, respectively. The percentages of G+C content are 58.4%, 58.6%, 57.4%, and 54.6% for the circular chromosome (scaffold 1), linear chromosome (scaffold 2), scaffold 3, and scaffold 4, respectively. A total of 5,718 putative coding sequences were predicted using the Rapid Annotations using Subsystems Technology (RAST) v4.0 automated pipeline (8). A BLAST search using scaffold 3 and scaffold 4 as queries indicated that both should be part of a plasmid related to the At plasmid of *A. tumefaciens* strain C58 encoding the lactonase BlcC.

### Nucleotide sequence accession numbers. 

The *A. tumefaciens* WRT31 genome sequence has been deposited at DDBJ/EMBL/GenBank under the accession no. APLP00000000. The version described in this paper is the first version, APLP01000000.
